# Implementation of Video Consultations Within a Personalized Hybrid Care Model for Children and Adolescents with Type 1 Diabetes Using Automated Insulin Delivery Systems: A Real-World Descriptive Study

**DOI:** 10.3390/jpm16070364

**Published:** 2026-07-04

**Authors:** Isolina Riaño-Galan, Corsino Rey, María Bogaerts Marquez, Laura Muñoz, Rebeca García, César Bazó, Julián Rodríguez

**Affiliations:** 1 Pediatrics Department, Central University Hospital of Asturias, 33011 Oviedo, Spain; crey@uniovi.es (C.R.); rebeca.garcia@sespa.es (R.G.); rodriguezjulian@uniovi.es (J.R.); 2 Research Institute of the Principality of Asturias (ISPA), 33011 Oviedo, Spain; maria.bogaerts@ispasturias.es; 3 Pediatrics Area, Department of Medicine, University of Oviedo, 33006 Oviedo, Spain; 4 Primary Care Interventions to Prevent Maternal and Child Chronic Diseases of Perinatal and Developmental Origin (RICORS-SAMID), Instituto de Salud Carlos III, 28029 Madrid, Spain; 5 Health Service of the Principality of Asturias, 33001 Oviedo, Spain; laura.munoz@sespa.es; 6 Teaching Commission, Central University Hospital of Asturias, 33011 Oviedo, Spain; cesar.bazo@sespa.es

**Keywords:** video consultation, telemedicine, personalized medicine, pediatric type 1 diabetes, continuous glucose monitoring, automated insulin delivery, hybrid care model, digital health

## Abstract

**Background:** Telemedicine complements traditional healthcare delivery and may improve access, continuity of care, and patient engagement, particularly in chronic conditions requiring regular follow-up. Video consultation is a widely adopted telemedicine modality and is increasingly integrated into hybrid care models. **Methods:** This real-world implementation project describes scheduled video consultations embedded in a hybrid care model for children and adolescents with type 1 diabetes using continuous glucose monitoring (CGM) and integrated insulin delivery technologies as part of routine clinical care. A total of 38 families were offered video consultations as part of routine care; 18 adopted the hybrid model. Video consultations were used for routine follow-up, shared review of device data, treatment adjustment, and diabetes education. Family experience was assessed using a voluntary 5-point Likert-scale satisfaction questionnaire. Complete longitudinal CGM data were available for 13 participants, all of whom were established users of the same automated insulin delivery (AID) platform (MiniMed™ 780G (Medtronic MiniMed, Inc. Minneapolis, MN, USA) integrated with Guardian™ 4 (Medtronic MiniMed, Inc. Minneapolis, MN, USA) continuous glucose monitoring). **Results:** Between 2022 and 2024, 162 video consultations were conducted. Acceptability was high, with 95% (17/18) of respondents reporting high satisfaction (score ≥ 4 on the 5-point Likert scale). 89% (16/18) of families perceived the quality of care as comparable to face-to-face visits for routine follow-up. Families highlighted convenience, reduced travel burden, and flexibility, as well as the value of shared review of CGM and AID system data. Group-level CGM-derived metrics appeared descriptively similar across sequential face-to-face visits and video consultations. Individual patient trajectories showed expected variability but no consistent pattern of deterioration during periods of remote follow-up. **Conclusions:** Video consultation is a feasible and well-accepted complementary modality within hybrid care models for pediatric type 1 diabetes. When integrated with CGM and automated insulin delivery systems, it supports personalized, data-driven clinical decision-making and continuity of care. Structured implementation and systematic evaluation are essential for sustainable integration into routine practice.

## 1. Introduction

Telemedicine has become a key component of contemporary healthcare delivery, enabling remote medical care through digital technologies that complement traditional face-to-face services. These approaches aim to improve access, continuity, and efficiency of care, particularly in chronic diseases that require long-term follow-up and close interaction between patients and healthcare professionals [1,2,3,4]. Recent advances in digital health have accelerated the integration of telemedicine into routine clinical practice, moving beyond isolated pilot experiences toward more structured care models.

Among telemedicine modalities, video consultation has emerged as one of the most widely adopted applications. It allows for real-time interaction, shared review of clinical data, and collaborative decision-making, while maintaining the option of face-to-face care when physical examination or in-person assessment is required. Video consultations are increasingly positioned as a core component of hybrid care models, combining in-person and remote follow-up [5]. Evidence suggests that this approach can enhance patient satisfaction, continuity of care, and engagement, especially in the management of chronic conditions [6,7].

Type 1 diabetes is a paradigmatic chronic disease that may particularly benefit from telemedicine-based follow-up. Pediatric diabetes care requires frequent treatment adjustments, continuous monitoring, and sustained involvement of patients and families. In this context, video consultations are increasingly used for routine follow-up, treatment adjustment, and therapeutic education, particularly when clinical decision-making relies primarily on the interpretation of digital data rather than on physical examination.

The widespread use of continuous glucose monitoring (CGM) in children and adolescents with type 1 diabetes has further expanded opportunities for personalized care. CGM provides real-time glucose data, enabling assessment of glycemic patterns, variability, and time in range, and supporting data-driven treatment decisions during both face-to-face visits and video consultations [8,9]. Many patients also use hybrid closed-loop or automated insulin delivery (AID) systems, which automatically adjust basal insulin delivery based on CGM data while meal-related boluses remain user-initiated. In the present implementation project, all participants included in the longitudinal analysis were users of the same AID platform (MiniMed™ 780G integrated with Guardian™ 4 CGM).

Beyond clinical complexity, pediatric diabetes care involves psychosocial challenges related to developmental stage, family dynamics, and the gradual transition toward self-management [10,11]. Hybrid care models combining face-to-face visits and video consultations may help reduce the burden of repeated hospital visits while maintaining continuity and quality of care and may also support family-centered and patient-centered approaches through digital tools that facilitate communication, education, and shared decision-making [12]. Emerging evidence from recent reviews suggests that telemedicine-based follow-up in children and adolescents with type 1 diabetes can support routine diabetes management with levels of care comparable to conventional follow-up, while improving accessibility, patient experience, and engagement. In some studies, telemedicine interventions have also been associated with favorable glycemic outcomes when appropriately integrated into standard care pathways [13,14].

Despite the increasing adoption of video consultations, real-world evidence describing their structured implementation within personalized pediatric diabetes care remains limited. This study aims to describe the implementation and outcomes of a video consultation project integrated into a hybrid care model for children and adolescents with type 1 diabetes using continuous glucose monitoring and integrated insulin delivery systems.

## 2. Materials and Methods

### 2.1. Study Design

This was a descriptive, observational, real-world implementation project evaluating the integration of video consultations into routine pediatric diabetes care.

### 2.2. Setting and Participants

The project was conducted in a pediatric diabetes outpatient clinic between 2022 and 2024. Participants were children and adolescents with type 1 diabetes followed at the clinic using continuous glucose monitoring (CGM) and integrated insulin delivery technologies as part of routine clinical care.

A total of 38 children and adolescents with type 1 diabetes and their families were offered the option of follow-up through video consultations as part of routine clinical care. Participation in video consultations was entirely voluntary, and families were explicitly allowed to choose their preferred model of care. Respect for patient and family preferences was a key component of the implementation strategy. Families were able to choose between hybrid follow-up and conventional face-to-face care according to their individual circumstances and preferences, consistent with principles of family-centered care and shared decision-making. Video consultation was offered as an alternative modality within standard follow-up, not as a mandatory intervention. Details of the diabetes technologies used by participants included in the longitudinal CGM analysis are provided in Section 2.5 and Table 1.

### 2.3. Video Consultation Model

Video consultations were implemented as a complementary modality within a hybrid care model combining face-to-face visits and remote follow-up. Consultations focused on shared review of CGM and insulin delivery data, individualized treatment adjustment, and diabetes education. All video consultations were conducted using secure institutional platforms compliant with data protection and confidentiality standards.

The hybrid care model consisted of four scheduled follow-up visits per year, identical in number to the standard face-to-face care pathway. Families who chose the hybrid model attended three video consultations per year (approximately one every three months) and one annual face-to-face visit, during which physical examination, assessment of growth and pubertal development, and annual laboratory testing were performed. Families who opted for exclusive face-to-face care followed the same schedule, with four in-person visits per year.

The operational workflow of video consultations included:

(1) Before the visit, upload and review of CGM and AID system data;
(2) During the visit, shared review of digital data, clinical discussion, treatment adjustment, and diabetes education;
(3) After the visit, documentation, reinforcement of educational messages, and follow-up planning.

Video consultations were used exclusively for routine follow-up, treatment adjustment, and diabetes education. Situations requiring physical examination, initiation of new devices, or management of acute conditions were addressed through face-to-face visits. In addition, video consultations supported family-centered care and psychosocial support by improving flexibility, reducing travel burden, and facilitating communication in a familiar home environment.

### 2.4. Outcomes and Satisfaction Questionnaire

Family satisfaction and experience were assessed using a locally developed 5-point Likert scale questionnaire (1 = strongly disagree; 5 = strongly agree), employed as part of routine quality assessment in clinical care.

The questionnaire comprised 25 items (Q1–Q25) organised into five domains: Accessibility (2 items: Q1–Q2), addressing ease of access to care and travel burden reduction; Quality of Medical Care (7 items: Q3–Q4, Q17–Q21), covering perceived equivalence to face-to-face visits, privacy, time adequacy, and continuity of care; Ease of Use and Technical Reliability (6 items: Q5–Q7, Q14–Q16), assessing usability, technical reliability, and error resolution; Physician–Patient Interaction (6 items: Q8–Q13), evaluating comfort, clarity of communication, and mutual understanding; and Overall Satisfaction and Future Use (4 items: Q22–Q25), capturing overall acceptability and intention to recommend. Responses were rated on a 5-point Likert scale (1 = strongly disagree; 5 = strongly agree).

Prior to its implementation, the questionnaire underwent an expert review process focused on content relevance, clarity and comprehensibility, involving 10 experts in pediatric care and digital health. Feedback from this pilot phase was used to refine item wording and structure.

The questionnaire was not intended as a standardized psychometric instrument, and no formal reliability or construct validity testing was performed. The complete questionnaire is provided as Supplementary Questionnaire S1 to enhance transparency and reproducibility. In addition, the questionnaire included an optional open-ended question (“Additional comments”), which allowed families to provide qualitative feedback. These comments were not included in the quantitative analysis and were used descriptively to illustrate participants’ experiences.

Violin plots were used exclusively for graphical visualization of response patterns. No statistical inferences were derived from these figures.

### 2.5. Descriptive Analysis of Glycemic Metrics

In addition, descriptive longitudinal analysis of CGM-derived glycemic metrics was performed during sequential face-to-face visits and video consultations within the hybrid care model. Evaluated parameters included time in range (TIR) (70–180 mg/dL), time above range (TAR), time below range (TBR), coefficient of variation (CV), and glucose management indicator (GMI). These data were analyzed descriptively without inferential statistical comparisons because of the exploratory nature of the study and the limited sample size.

Complete longitudinal data across the predefined sequence of five consultations were available for 13 participants. All participants included in this longitudinal analysis used the same automated insulin delivery platform (MiniMed™ 780G integrated with Guardian™ 4 continuous glucose monitoring) and had been using the system for at least six months before study entry.

### 2.6. Ethical Considerations

Video consultations and satisfaction surveys were conducted as part of routine clinical care and quality assessment and did not involve any interventional research procedures. No personal or identifiable data were collected. Data derived from the voluntary family satisfaction survey were fully anonymized and analyzed in aggregated form.

The study protocol, including the secondary analysis and scientific dissemination of fully anonymized aggregated data derived from routine clinical care and quality assessment, was reviewed and approved by the Research Ethics Committee for Medicines of the Principality of Asturias (CEImPA; approval reference 2025.538), which granted a waiver of written informed consent.

## 3. Results

### 3.1. Participant Flow and Use of Video Consultations

Of the 38 families offered the option of video consultations, 12 declined due to practical limitations such as lack of stable internet connection or appropriate devices, and 4 declined because they preferred exclusive face-to-face care. An additional 4 families initially tried one video consultation but subsequently chose to return to face-to-face follow-up. A total of 18 families adopted the hybrid care model and were included in the implementation project and satisfaction survey.

### 3.2. Implementation of Video Consultations

Between 2022 and 2024, a total of 162 scheduled video consultations were conducted among the 18 families who adopted the hybrid care model. The median age of the patients was 15 years (range: 5–16 years).

### 3.3. Baseline Characteristics of the Longitudinal Cohort

Baseline demographic and clinical characteristics of the 13 participants included in the longitudinal CGM analysis are summarized in Table 1. The cohort had a mean age of 13.3 ± 2.6 years and a mean diabetes duration of 7.5 ± 3.1 years. Nine participants (69.2%) were female. Baseline glycemic control was generally good, with a mean TIR of 77.0 ± 6.5%, mean GMI of 6.79 ± 0.28%, and mean HbA1c of 6.83 ± 0.31%. Baseline values correspond to the first scheduled face-to-face consultation.

All participants included in the longitudinal analysis used the same automated insulin delivery platform (MiniMed™ 780G integrated with Guardian™ 4 continuous glucose monitoring) and had been using the system for at least six months before study entry.

### 3.4. Acceptability and Satisfaction

Satisfaction questionnaire responses reflect families’ overall experience with video consultations rather than individual visits. To avoid issues related to lack of independence of observations, repeated consultations were not treated as independent units, and no inferential statistical analyses were performed.

Overall acceptability of video consultations was high. As summarized in Table 2, 95% (17/18) of families reported high satisfaction, highlighting convenience, reduced travel time, and increased flexibility. No relevant technical errors were experienced in 78% (14/18) of consultations; when technical issues occurred, two out of every 3 were easily resolved.

Qualitative comments derived from the optional open-ended question were used illustratively to complement the quantitative findings. Families emphasized time savings, reduced disruption of school and work activities, and overall convenience. Representative comments included:

“As a family where both of us work, it has saved us a lot of time, not having to go to the hospital in person, and my son also didn’t have to miss so much school time” and “Everything was perfect, a very convenient consultation and saving the time spent traveling. I would repeat without hesitation.”

### 3.5. Perceived Quality of Care

Perceived quality of care delivered through video consultations was comparable to face-to-face visits for routine follow-up. Overall, 89% (16/18) of families considered the quality-of-care equivalent to in-person consultations. In addition, 83% (15/18) reported feeling comfortable during video consultations, particularly highlighting the clarity of visual and auditory communication. One parent noted: “I was able to talk to our doctor even better than during the face-to-face consultation.”

### 3.6. Clinical and Educational Aspects of Routine Care

Video consultations supported shared review of CGM and AID system data during routine follow-up, allowing discussion of glycemic patterns and treatment decisions. Families reported improved understanding of device data and greater involvement in diabetes self-management.

Video consultations also facilitated participation of multiple family members despite geographical separation. In some cases, adolescents attended consultations directly from their school setting, thereby avoiding school absenteeism, while both parents were able to participate simultaneously from different workplaces or even from another European country despite geographical separation. This flexibility supported family involvement and shared decision-making without requiring all participants to be physically present in the same location.

### 3.7. Evolution of Glycemic Metrics During Hybrid Follow-Up

To explore glycemic evolution during the hybrid care pathway, CGM-derived metrics obtained during sequential face-to-face visits and video consultations were descriptively analyzed. Evaluated parameters included TIR 70–180 mg/dL, TAR, TBR, CV, and GMI.

Complete longitudinal CGM-derived data across all five sequential consultations were available for 13 of the 18 participating families. In the remaining participants, complete datasets could not be retrieved due to device changes during follow-up, missing data uploads at specific visits, or incomplete hybrid follow-up sequences related to the voluntary nature of participation. No systematic pattern of missing data was identified.

As specified in the Methods section, no inferential statistical analyses were performed. Accordingly, the data are presented descriptively to provide contextual information regarding the glycemic profile of the cohort during hybrid follow-up.

**Figure 1:**
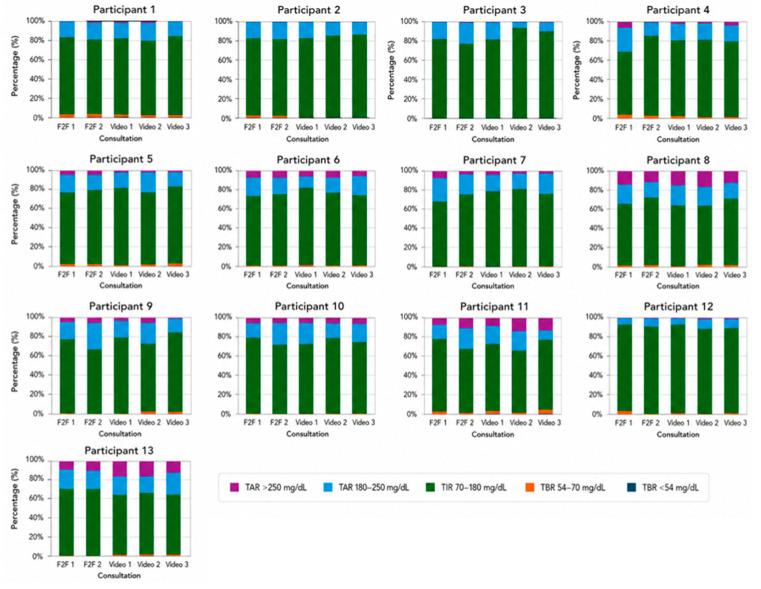
Individual longitudinal distribution of CGM-derived glycaemic metrics across five sequential consultations in participants with complete follow-up (*n* = 13).

**Figure 2:**
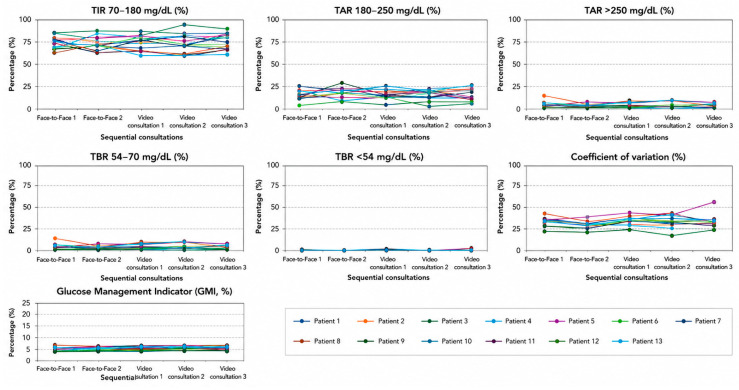
Individual longitudinal trajectories of CGM-derived glycaemic metrics during hybrid follow-up.

Table 3 summarizes the mean ± standard deviation of CGM-derived glycaemic metrics across the five predefined consultations. Group-level values appeared descriptively similar throughout follow-up. Figure 1 presents the individual longitudinal glycaemic profiles of participants with complete follow-up, illustrating the proportional distribution of time spent within each glycaemic range across sequential consultations. Complementing these profiles, Figure 2 displays the longitudinal trajectories of each CGM-derived metric, highlighting the expected inter-individual variability observed in routine clinical practice without revealing a consistent pattern of deterioration during periods of remote follow-up.

When assessed against the internationally accepted CGM targets proposed by Battelino et al. [9], all key glycemic parameters remained within recommended ranges throughout both face-to-face visits and video consultations.

Individual patient trajectories demonstrated heterogeneous but generally stable patterns of glycemic control throughout the hybrid follow-up sequence, without evidence of marked deterioration during remote follow-up periods.

To improve transparency and address the potential limitations of relying solely on group-level summary statistics, individual longitudinal graphical representations of CGM-derived metrics were incorporated (Figure 1 and Figure 2). These figures allow visualization of within-patient variability and complement the descriptive data presented in Table 3. 

Each panel represents one participant and displays the proportional distribution of continuous glucose monitoring (CGM)-derived glycaemic metrics across the predefined sequence of two face-to-face consultations (F2F1 and F2F2) followed by three scheduled video consultations (Video 1–3). Stacked bars represent the percentage of time spent in each glycaemic range: time in range (TIR, 70–180 mg/dL), time above range (TAR, 180–250 mg/dL and >250 mg/dL), and time below range (TBR, 54–70 mg/dL and <54 mg/dL). The figure illustrates individual longitudinal glycaemic profiles and the expected inter-individual variability observed during hybrid follow-up, complementing the descriptive summary statistics presented in Table 3.

Individual patient trajectories are shown for time in range (TIR 70–180 mg/dL), time above range (TAR 180–250 mg/dL and >250 mg/dL), time below range (TBR 54–70 mg/dL and <54 mg/dL), coefficient of variation (CV), and glucose management indicator (GMI) across the predefined sequence of two face-to-face visits and three scheduled video consultations. The figures are presented to illustrate within-patient variability over time and complement the descriptive summary statistics reported in Table 3. Owing to the descriptive design of the study and the limited sample size, these graphical representations should be interpreted as exploratory visualizations rather than inferential analyses.

### 3.8. Safety Observations During Hybrid Follow-Up

No episodes of acute diabetes-related decompensation requiring hospital admission were recorded among the 18 participants who adopted the hybrid video consultation model during the implementation period. Given the descriptive nature of the project and the absence of a predefined comparator group, no comparative analyses were performed. This observation should be interpreted descriptively and not as evidence of improved safety compared with conventional care.

### 3.9. Distribution of Survey Responses

To complement the descriptive summary of questionnaire responses, violin plots were used as an exploratory graphical visualization of the distribution of Likert-scale scores across questionnaire domains and individual items. Given the small sample size and the ordinal nature of the data, these figures are intended solely to facilitate visual inspection of response patterns and should not be interpreted as estimates of continuous probability distributions.

Violin plots show the distribution of responses across five domains of the family satisfaction questionnaire: Accessibility (Q1–Q2), Quality of Medical Care (Q3–Q4, Q17–Q21), Ease of Use and Technical Reliability (Q5–Q7, Q14–Q16), Physician–Patient Interaction (Q8–Q13), and Overall Satisfaction (Q22–Q25). Individual dots represent responses from participating families. Responses were rated on a 5-point Likert scale ranging from 1 (strongly disagree) to 5 (strongly agree), with higher scores indicating greater agreement or satisfaction.

**Figure 3:**
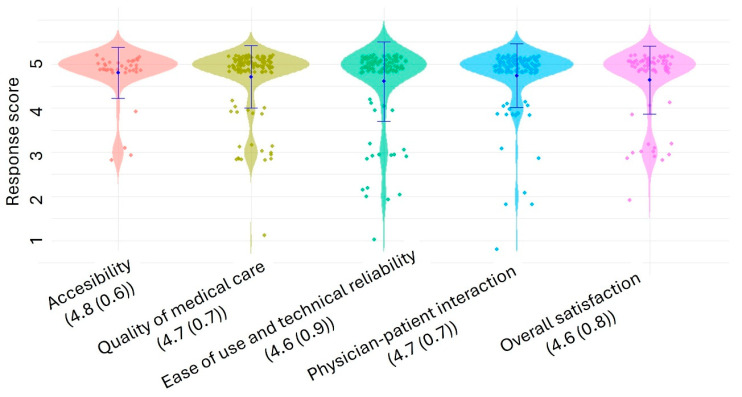
Exploratory visualization of family satisfaction scores across questionnaire domains.

Figure 3 provides an exploratory graphical visualization of the distribution of family satisfaction scores across the five questionnaire domains.

Violin plots are presented as an exploratory graphical display of the distribution of Likert-scale responses across the five questionnaire domains. Individual dots represent responses from participating families (*n* = 18). Because responses were measured on a five-point ordinal scale and the sample size was small, the violin shapes should be interpreted only as a visual aid to illustrate overall response patterns rather than as estimates of continuous data distributions.

**Figure 4:**
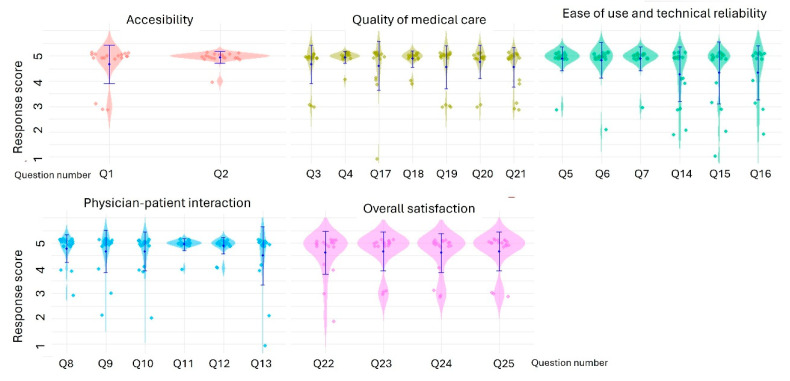
Distribution of family-reported survey responses across domains of video consultation experience.

Figure 4 complements these results by displaying the distribution of responses for each individual questionnaire item grouped by domain.

Violin plots illustrate the distribution of responses to individual questionnaire items (Q1–Q25), grouped by domain: Accessibility (Q1–Q2), Quality of Medical Care (Q3–Q4, Q17–Q21), Ease of Use and Technical Reliability (Q5–Q7, Q14–Q16), Physician–Patient Interaction (Q8–Q13), and Overall Satisfaction (Q22–Q25). Individual dots represent responses from participating families. Responses were rated on a 5-point Likert scale ranging from 1 (strongly disagree) to 5 (strongly agree).

## 4. Discussion

This study shows that video consultation can be feasibly integrated into a hybrid care model for pediatric type 1 diabetes, supporting personalized, data-informed clinical management while complementing face-to-face visits rather than replacing them.

The high levels of acceptability and satisfaction observed in this real-world project are consistent with previous evidence on telemedicine in pediatric diabetes. A recent systematic review and meta-analysis by Zhang et al. reported that telemedicine-based follow-up in children and adolescents with type 1 diabetes can achieve levels of glycemic control comparable to conventional care and, in some settings, may be associated with improvements in glycemic outcomes, while also enhancing accessibility and continuity of care [14]. Similarly, Matter et al. described a positive impact of telecommunication services on quality of life and continuity of care for children and adolescents with type 1 diabetes during and after the COVID-19 pandemic [15].

A key strength of the model described lies in its integration with advanced diabetes technologies. Recent consensus recommendations identify automated insulin delivery (AID) systems as the current standard of care in pediatric diabetes, emphasizing that their safe and effective use depends on continuous expert review of device-generated data [8]. Video consultation facilitates a collaborative environment for expert review of device-generated data, supporting individualized treatment decision-making within routine clinical care. In our real-world cohort, group-level CGM-derived glycemic metrics appeared descriptively similar throughout the hybrid follow-up pathway. Although individual patient trajectories showed the expected variability of routine clinical practice, no consistent pattern of short-term deterioration in glycemic control was observed during periods of remote follow-up. These descriptive findings are compatible with the maintenance of continuity in data-driven diabetes management within the hybrid care model but should not be interpreted as evidence of clinical effectiveness. This approach is consistent with real-world evidence demonstrating that sustained glycemic outcomes depend on structured clinical supervision [10].

Importantly, participants included in the longitudinal analytical cohort were experienced users of the MiniMed™ 780G–Guardian™ 4 automated insulin delivery platform and had been using the system for at least six months before study entry. Consequently, the observed glycemic patterns are unlikely to reflect early adaptation to the technology itself. The technological homogeneity of the cohort also reduced variability attributable to differences between diabetes devices, facilitating interpretation of the descriptive longitudinal findings.

Beyond clinical management, video consultation also supports patient- and family-centered care, shared decision-making, and psychosocial support, which are particularly relevant in pediatric diabetes [11,12]. Improved flexibility, reduced travel burden, and the possibility of engaging families in a familiar home environment may contribute positively to the perceived overall care experience.

An important strength of this implementation project was the preservation of patient and family choice regarding the preferred model of follow-up. Rather than promoting a single mode of care, families were offered the opportunity to select the follow-up strategy that best matched their preferences and circumstances. In this sense, personalization extended beyond the use of advanced diabetes technologies and included adaptation of healthcare delivery to individual family needs.

An additional advantage observed during implementation was the possibility of involving adolescents and multiple family members simultaneously despite geographical separation. Adolescents were sometimes able to participate directly from their school setting, avoiding school absenteeism, while parents joined consultations from different workplaces or even from another European country. This flexibility facilitated participation in diabetes management discussions, promoted family-centered care, and strengthened shared decision-making without requiring all participants to be physically present in the same location.

No diabetes-related hospital admissions were recorded among participants who adopted the hybrid model during follow-up. Although this observation may be reassuring from a safety perspective, the study was not designed to compare acute-event rates and no conclusions regarding effectiveness or risk reduction can be drawn.

**Figure 5:**
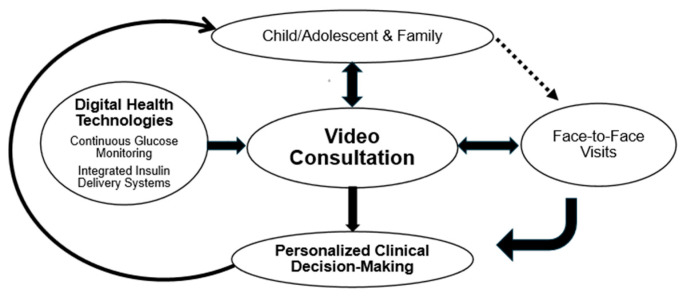
Hybrid care model for pediatric type 1 diabetes using video consultation.

From an implementation perspective, the findings of this project align with expert consensus recommendations on telemedicine management of diabetes [16] and with international digital health frameworks from the World Health Organization and the Pan American Health Organization, which emphasize the importance of structured implementation, monitoring, and evaluation of digital health interventions [17,18]. As illustrated in Figure 5, video consultation was integrated into a hybrid care model combining face-to-face visits and digital health technologies, including continuous glucose monitoring and integrated insulin delivery systems. This model positions video consultation as a complementary modality within established pediatric diabetes care pathways, supporting shared data review, personalized clinical decision-making, and continuity of care.

Video consultation is integrated into a hybrid care model combining face-to-face visits and digital health technologies, including continuous glucose monitoring and integrated insulin delivery systems. Collaborative review of digital data supports individualized clinical decision-making, continuity of care and patient-centered management in children and adolescents with type 1 diabetes.

From a practical implementation perspective, the experience gained during this project suggests that scheduled video consultations can facilitate routine follow-up when clinical decision-making is primarily based on the interpretation of remotely available CGM and automated insulin delivery data rather than on physical examination. Their integration within a structured hybrid care pathway allowed remote consultations to be reserved for situations in which in-person assessment was not clinically required. The flexibility of this model also enabled greater participation of adolescents and family members in routine consultations, illustrating how hybrid care can be adapted to the practical circumstances of families while maintaining continuity of specialist follow-up.

Nevertheless, consistent with existing evidence and clinical experience, video consultation is not appropriate for all clinical situations. Circumstances requiring physical examination, device initiation, or resolution of complex technical issues continue to benefit from face-to-face assessment. These considerations further reinforce the role of video consultation as a complementary component within hybrid care models.

Several limitations should be acknowledged. This study has a descriptive design without a control group, and the findings should therefore be interpreted as indicators of feasibility and acceptability rather than evidence of clinical effectiveness. Although descriptive CGM-derived glycemic metrics were included, the study was not designed or powered to formally evaluate clinical effectiveness. No inferential statistical analyses were planned or performed because of the exploratory nature of the project, the absence of a comparator group, and the limited number of participants with complete longitudinal data. Accordingly, CGM-derived metrics were presented descriptively to provide contextual information regarding glycemic patterns during hybrid follow-up. The satisfaction questionnaire was a locally developed instrument that underwent expert review for content relevance, clarity and comprehensibility but was not subjected to formal psychometric validation. In addition, outcomes were primarily self-reported and may be influenced by social desirability and Hawthorne effects. The voluntary nature of participation and the small sample size raise the possibility of selection bias, and repeated video consultations per patient were not treated as independent observations. The graphical representation of questionnaire responses using violin plots was intended exclusively as an exploratory visualization to facilitate interpretation of response patterns. Given the ordinal nature of Likert-scale data and the limited sample size, these figures should not be interpreted as formal density estimates or inferential statistical analyses.

Despite these limitations, the real-world nature of the project provides valuable insights into the integration of video consultations into routine pediatric diabetes care. A notable limitation of this project is the absence of systematic data on the 20 families who did not adopt the hybrid care model. Of these, 12 declined due to practical barriers such as lack of stable internet access or appropriate devices, 4 preferred exclusive face-to-face follow-up, and 4 initially participated in one video consultation before returning to in-person care. Although these proportions provide some insight into the reasons for non-adoption, no structured data were collected on the characteristics of non-adopters (e.g., patient age, educational level, duration of diabetes, or type of insulin delivery system used). This limits our ability to characterize the population most likely to benefit from or engage with hybrid models and precludes any comparison between adopters and non-adopters. Future implementation studies should prospectively collect standardized data from both adopters and non-adopters to better characterize barriers and facilitators of hybrid care implementation and to inform strategies that promote equitable access to telemedicine services.

All participants included in the longitudinal CGM analysis were established users of the same advanced automated insulin delivery platform (MiniMed™ 780G with Guardian™ 4). While this technological homogeneity reduced variability attributable to device-related factors, it may limit the generalizability of the findings to patients using other diabetes technologies or less technology-intensive treatment regimens.

Furthermore, because participation in the hybrid model was voluntary, the study population likely represents families with greater willingness to engage in telemedicine-based follow-up. Consequently, the findings should be interpreted as applying primarily to self-selected adopters of hybrid care.

## 5. Conclusions

Video consultation integrated into a hybrid care model appears to be a feasible and well-accepted complementary approach to pediatric type 1 diabetes management. When combined with continuous glucose monitoring and automated insulin delivery systems, video consultation is a well-accepted tool for routine follow-up, supporting personalized, data-informed clinical decision-making, continuity of care, and patient- and family-centered management.

In this real-world implementation project, group-level CGM-derived metrics appeared descriptively similar throughout sequential face-to-face visits and video consultations. Individual patient trajectories demonstrated expected variability but no consistent pattern of deterioration during periods of remote follow-up. Given the descriptive design, small sample size, and absence of a comparator group, no conclusions regarding clinical effectiveness can be drawn.

Consistent with expert consensus recommendations and international digital health frameworks, structured implementation and systematic evaluation are essential to ensure the sustainable integration of video consultations into routine pediatric diabetes care.

Future prospective studies with larger samples, controlled designs, and the inclusion of objective clinical endpoints are needed to establish the effectiveness of video consultation-based hybrid care models and to identify the patient profiles that may benefit most from this approach.

## Figures and Tables

**Table 1:** Baseline characteristics of participants included in the longitudinal CGM analysis (*n* = 13).

Characteristic	Value
Age, years	13.3 ± 2.6 (9–17)
Female sex, *n* (%)	9 (69.2)
Diabetes duration, years	7.5 ± 3.1 (3–13)
Age at diabetes onset, years	5.8 ± 2.0 (2–10)
Baseline TIR 70–180 mg/dL, %	77.0 ± 6.5 (65–88)
Baseline GMI, %	6.79 ± 0.28 (6.3–7.3)
HbA1c, %	6.83 ± 0.31 (6.2–7.4)
Coefficient of variation (CV), %	32.9 ± 4.4 (25–41)
MiniMed™ 780G use, n (%)	13 (100)
Guardian™ 4 CGM use, n (%)	13 (100)
Automated insulin delivery (AID), n (%)	13 (100)
Duration of AID use before study entry	≥6 months in all participants

Data are presented as mean ± standard deviation (range), unless otherwise indicated. All participants were established users of the same automated insulin delivery platform (MiniMed™ 780G integrated with Guardian™ 4 continuous glucose monitoring) and had been using the system for at least six months before study entry. Abbreviations: TIR, time in range; GMI, glucose management indicator; HbA1c, glycated haemoglobin; CV, coefficient of variation; CGM, continuous glucose monitoring; AID, automated insulin delivery.

**Table 2:** Summary of results of the video consultation implementation project.

Domain	Outcome	Results
Implementation	Video consultations	162 consultations (2022–2024)
Care model	Hybrid care model	Face-to-face visits + video consultations
Population	Target group	Children and adolescents with type 1 diabetes
Technologies	Devices used	CGM and integrated insulin delivery systems
Accessibility	Ease of access and reduced travel	High agreement (Q1–Q2)
Quality of Medical Care	Perceived quality vs face-to-face	89% (16/18) perceived as comparable (Q3–Q4, Q17–Q21)
Ease of Use and Technical Reliability	System usability	High agreement (Q5–Q7, Q14–Q16)
Physician–Patient Interaction	Communication quality	High agreement (Q8–Q13)
Overall Satisfaction	Satisfaction and future use	95% (17/18) highly satisfied (Q22–Q25)

Abbreviations: CGM, continuous glucose monitoring.

**Table 3:** Evolution of CGM-derived glycaemic metrics during hybrid follow-up (*n* = 13).

Metric	Face-to-Face 1	Video 1	Video 2	Video 3	Face-to-Face 2
TIR 70–180 mg/dL (%)	77.0 ± 6.5	77.0 ± 7.5	76.6 ± 9.6	78.4 ± 8.1	76.7 ± 6.5
TAR 180–250 mg/dL (%)	17.2 ± 5.3	17.0 ± 4.8	17.4 ± 5.5	16.5 ± 5.7	19.6 ± 5.4
TAR > 250 mg/dL (%)	3.8 ± 2.8	4.2 ± 3.4	4.5 ± 4.3	3.5 ± 2.7	3.0 ± 2.4
TBR 54–70 mg/dL (%)	2.0 ± 1.7	1.5 ± 1.3	1.4 ± 0.8	1.3 ± 1.3	0.8 ± 0.8
TBR < 54 mg/dL (%)	0.1 ± 0.3	0.3 ± 0.6	0.2 ± 0.4	0.2 ± 0.6	0.0 ± 0.0
CV (%)	32.9 ± 4.4	33.7 ± 4.5	32.3 ± 6.4	33.5 ± 8.0	29.6 ± 4.1
GMI (%)	6.8 ± 0.3	6.8 ± 0.3	6.9 ± 0.3	6.8 ± 0.3	6.9 ± 0.2

Data are presented as mean ± standard deviation. Values correspond to the 13 participants with complete longitudinal continuous glucose monitoring (CGM) data across the predefined sequence of five consultations (two face-to-face visits and three scheduled video consultations). Owing to the descriptive design of the study, the absence of a comparator group, and the limited sample size, no inferential statistical comparisons were performed. Individual patient trajectories are presented in Figure 2 to complement these summary statistics. Abbreviations: CGM, continuous glucose monitoring; TIR, time in range; TAR, time above range; TBR, time below range; CV, coefficient of variation; GMI, glucose management indicator.

## Data Availability

The data presented in this study are based on anonymized and aggregated information collected as part of routine clinical quality assessment. Individual-level data are not publicly available due to ethical and privacy considerations. The satisfaction questionnaire used in the study is provided as Supplementary Material (Supplementary Material S1).

## Supplementary Materials

The following supporting information can be downloaded at https://www.mdpi.com/article/10.3390/jpm16070364/s1. Supplementary Questionnaire S1: Family satisfaction questionnaire used to assess parents’ perceptions of the hybrid video consultation model.

## Abbreviations

The following abbreviations are used in this manuscript:

CGM	Continuous glucose monitoring
AID	Automated insulin delivery
TIR	Time in range
TAR	Time above range
TBR	Time below range
CV	Coefficient of variation
GMI	Glucose management indicator
CEImPA	Research Ethics Committee for Medicines of the Principality of Asturias
