# Supplementary material for: Implementation of Video Consultations Within a Personalized Hybrid Care Model for Children and Adolescents with Type 1 Diabetes Using Automated Insulin Delivery Systems: A Real-World Descriptive Study
Source: J Pers Med. 2026 Jul 4;16(7):364. doi: 10.3390/jpm16070364 (PMC13412691; doi:10.3390/jpm16070364)
Supplement: Supplementary file 1 [file jpm-16-00364-s001.zip › jpm-4371926-supplementary.pdf]

Supplementary Material S1.

Family satisfaction questionnaire used to assess parents' perceptions of the hybrid video consultation model

This questionnaire was developed for routine quality assessment of the hybrid video consultation programme in the Paediatric Diabetes Unit. It was reviewed by experts for content relevance, clarity and comprehensibility and was administered to participating families following implementation of the hybrid care model.

Introduction

Dear patient / Dear family,

We would like to learn about your experience with the video consultations you have received. This survey is intended for patients and families who have already participated in several video consultations and aims to help us improve the quality of the care we provide. Your responses are very valuable and will help us improve our service.

Please answer the following questions based on your **overall experience** with the video consultation system.

Response scale:
Responses are rated on a 5-point Likert scale, where **1 = strongly disagree** and **5 = strongly agree**.

Accessibility (Q1–Q2)

Q1. Video consultations make it easier for me to access healthcare services.
Q2. Video consultations save me travel time to the hospital or healthcare center.

Quality of Medical Care (Q3–Q4, Q17–Q21)

Q3. Video consultations meet my healthcare needs.
Q4. I have received appropriate care using the video consultation system.

Q17. I believe that the care provided through video consultations has been similar to that provided during an in-person consultation.
Q18. The time allocated for the video consultation has been sufficient to meet

my needs.
Q19. I feel that my privacy is respected when using the video consultation system.
Q20. My doctor uses the information obtained during video consultations in subsequent in-person visits.
Q21. The lack of physical contact during video consultations is not a problem for me.

Ease of Use and Technical Reliability (Q5–Q7, Q14–Q16)

Q5. The system has been easy for me to use.
Q6. The instructions provided by the healthcare team for using the system have been adequate and sufficient.
Q7. I believe I could easily adapt to using this system.

Q14. I feel that the system is reliable (not prone to errors).
Q15. There have been no computer or technical errors that hindered the process.
Q16. In case of a technical error, it has been easy to resolve it.

Physician–Patient Interaction (Q8–Q13)

Q8. I have felt comfortable talking to my doctor during video consultations.
Q9. I have been able to hear my doctor clearly.
Q10. I have been able to speak clearly with my doctor.
Q11. I have been able to express myself without any difficulties.
Q12. My doctor has been able to understand my health condition.
Q13. I have been able to see my doctor clearly, similar to an in-person consultation.

Overall Satisfaction and Future Use (Q22–Q25)

Q22. Video consultations are an acceptable way to receive medical care.
Q23. I would like to use video consultation services again.
Q24. I would recommend the use of video consultation services to others.
Q25. Overall, I am satisfied with the quality of the video consultation service.

Open-Ended Question (Optional)

Additional comments:

Please use the space below to share any additional comments, suggestions, or experiences related to your use of video consultations that you consider relevant.

This open-ended question was optional and was not included in the quantitative analysis. Responses were used qualitatively to illustrate families' experiences.
